# Considering Opportunities and Challenges When Implementing the Model Master File Framework – a Meeting Report

**DOI:** 10.1007/s11095-025-03839-x

**Published:** 2025-03-07

**Authors:** Eleftheria Tsakalozou, Lanyan Fang, Erin Skoda, Timothy Nicholas, Sivacharan Kollipara, Ke Ren, Stella Grosser, Bhagwant Rege, Partha Roy, Hao Zhu, Liang Zhao

**Affiliations:** 1 Division of Quantitative Methods and Modeling, Office of Research and Standards (ORS), Office of Generic Drugs (OGD), Center for Drug Evaluation and Research (CDER), U.S. Food and Drug Administration (FDA), Silver Spring, MD 20993 USA; 2 Office of Product Quality Assessment III, Office of Pharmaceutical Quality, Center for Drug Evaluation and Research (CDER), U. S. Food and Drug Administration (FDA), Silver Spring, MD USA; 3 Pfizer Inc., Cambridge, MA USA; 4 Biopharmaceutics Group, Global Clinical Management, Integrated Product Development Organization (IPDO), Dr. Reddy’s Laboratories Ltd, Bachupally, Medchal Malkajgiri District, Hyderabad, 500 090 Telangana India; 5 Office of Bioequivalence, Office of Generic Drugs, Center for Drug Evaluation and Research, U.S. Food and Drug Administration, Silver Spring, MD USA; 6 Office of Biostatistics, Center for Drug Evaluation and Research, U.S. Food and Drug Administration, Silver Spring, Maryland, USA; 7 Office of Product Quality Assessment I, Office of Pharmaceutical Quality (OPQ), Center for Drug Evaluation and Research (CDER), U.S. Food and Drug Administration (FDA), Silver Spring, MD 20903-1058 USA; 8 Office of Clinical Pharmacology, Office of Translational Sciences, Center for Drug Evaluation and Research (CDER), U.S. Food and Drug Administration (FDA), Silver Spring, MD USA

**Keywords:** model master file, modeling and simulation, workshop report

## Abstract

**Supplementary Information:**

The online version contains supplementary material available at 10.1007/s11095-025-03839-x.

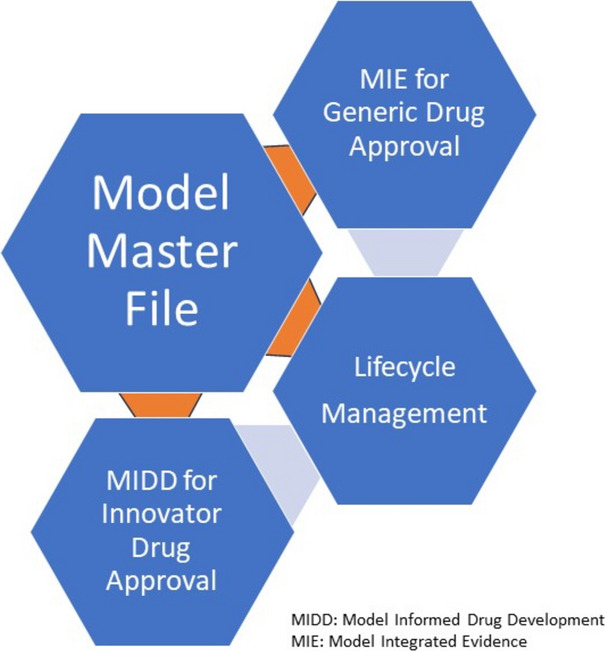

## Introduction

The impact of modeling and simulation approaches on decision-making for drug product development and regulatory approval is constantly increasing. Technological advancements that improve computational efficiency, scientific exchange within diversified communities that include clinical pharmacologists, statisticians, experimentalists, translational scientists and clinicians, and the development of roadmaps that seamlessly integrate modeling and simulation (M&S) in the drug development paradigm are supporting the critical role modeling and M&S is playing today in drug development. In the regulatory setting, acceptance of these in silico methodologies is constantly increasing as shown by general guidance’s on best practices for these methodologies are published by regulatory agencies and new and generic drug products are approved with the support of M&S applications [1–4].

In the realm of new drugs including biologics, model informed drug development (MIDD), including methodologies and tools, have supported multiple applications. These applications are spanning from predictions at very early stage of the drug development including candidate and formulation selection, to proposing a first-in-humans dose, informing clinical decisions at later stage of drug development such as assessing the potential of drug-drug interactions (DDIs), performing risk assessments and formulation bridging, supporting waivers of human testing, informing dosing selections in special populations such as pediatrics, liver and kidney impaired populations, addressing feasibility challenges for rare diseases, improving the biopharmaceutics profile of drug candidates, and addressing quality issues throughout the life cycle of a product and predicting therapeutic outcomes in virtual patients [2, 5–11]. Similarly, in the case of product development and lifecycle management, MIDD aided in plethora of applications to address, for example, dissolution specifications justification, critical bioavailability attributes identification & control, biowaiver of lower strengths and specific studies, and development of bioequivalent formulations [9, 10, 12–16].

In the realm of generic drugs, quantitative methods and modeling (QMM) such as quantitative clinical pharmacology models, physiologically based pharmacokinetic (PBPK) models, computational fluid dynamics (CFD) models and other in silico mechanistic models have modernized the development of generic drugs and supported regulatory assessments through a model integrated evidence (MIE) paradigm [1, 17–21]. These models are grounded on their capability to account for drug product specific properties to predict delivery of the active ingredient at or close to the site of action. When suitably verified and validated for their intended purpose, these models can leverage predictions reflecting formulation characteristics that may differ between a brand name product and its prospective generic to perform a virtual bioequivalence (BE) assessment. Physiologically based biopharmaceutics models (PBBM) that incorporate *in vitro* dissolution and other formulation characteristics, are applied by innovator companies and developers of generics towards quality assurance throughout the life cycle of products [10, 11, 20, 22].

In silico models supporting a specific regulatory application, such as the ones referenced previously, are typically the result of a rigorous development and documentation process by applicants. After submitted to regulatory agencies, they are subject to detailed scientific and regulatory assessment. With an aim to reduce applicants’ efforts in preparing these types of submissions and to reduce certain regulatory review timelines, FDA introduced the Model Master File (MMF) concept.

The MMF framework was first discussed in the 2021 public workshop co-hosted by FDA and the Center for Research on Complex Generics (CRCG): “Regulatory Utility of Mechanistic Modeling to Support Alternative Bioequivalence Approaches” and explored further in other public platforms [23, 24]. ​A recent publication by Fang *et al*., 2024 described an MMF as “a quantitative model or a modeling platform that has undergone sufficient model Verification & Validation [to be] recognized as sharable intellectual property that is acceptable for regulatory purposes” [25]. M&S applications that support regulatory approval or tentative approval were featured as potential MMF examples. These examples included: a validated modeling framework to increase credibility of models developed for products of the same route of administration, modeling methodologies such as model-based data imputation to handle missing data in the clinical setting and adequately validated models that support risk-based assessments on drug product quality and *in vivo* performance within the scope of product lifecycle management. A software platform that employs nonlinear mixed effects modeling supporting BE assessment of a drug with long half-life and a mechanistic PBBM that serves as the basis for the development of an *in vitro*-*in vivo* correlation (IVIVC) for an oral dosage form were discussed within the context of the conceptual MMF framework. Through these case studies and during interactions in public forums, the community recognized the potentially transformative nature of the MMF framework in streamlining the development and application of M&S in drug development leading to industry and regulatory acceptability of M&S approaches while considering potential unintended consequences.

To deepen the community’s understanding on the MMF initiative, the FDA co-hosted with the Center for Complex Generics (CRCG, https://www.complexgenerics.org) a hybrid public workshop titled “Considerations and Potential Regulatory Applications for a Model Master File” [26]. This report summarizes the podium presentations and panel discussion from the workshop’s Day 1/ Session 1: “Defining the MMF Framework: Model Sharing-Model Acceptance-Model Communication”. The objective of this session was to promote a thorough discussion on the impact the MMF framework can have on the current drug product development and approval paradigm. The workshop attendees considered the implementation of the MMF initiative within the already established regulatory mechanism of a Drug Master File (DMF). Importantly, the speakers and panelists engaged in an exchange on the benefits, unintended consequences, and challenges in the implementation of the MMF for innovator drugs as well as generics.

## Overall Summary

The session was moderated by Drs. Lanyan Fang and Ke Ren (FDA) and included four podium presentations delivered by FDA, innovator and generic drug industry speakers followed by a 30-min panel discussion during which the speakers were joined by five additional panelists from the FDA and the European Medicines Agency (EMA). The first two talks by Drs Liang Zhao and Erin Skoda were designed to improve the understanding on operational aspects, benefits and potential challenges associated with the implementation of MMFs under the same guiding principles as DMFs. Drs Nicholas and Kollipara discussed pathways that the MMFs can be integrated into the product development paradigm for innovator drugs and generics, respectively and support successful regulatory submission for these products.

Importantly, this report summarizes the small group discussion sessions that took place at the end of Day 1 and 2 of the workshop and are not available in the public domain. This in person portion of the workshop is available in the Supplementary Material and contains useful information on the development of the MMF content and operational aspects of the MMFs and discussions on benefits for pharmaceutical industry and regulatory agencies associated with implementing MMFs.

Take home messages from the podium presentations, the panel discussion and the small group discussion sessions include:

- An MMF may serve as a benchmark or standardization for in silico methodologies, tools and applications, promote the development of “best practices” and at the same time foster technological innovation.
- The “context of use" for an MMF should be clearly stated and closely considered when the same MMF is referenced across several regulatory submissions.
- The MMF framework holds great potential for advancing the role and acceptance of M&S approaches and improve accessibility to these methodologies by the pharmaceutical industry.
- From a regulatory standpoint, the MMF framework can increase regulatory assessment consistency and efficiency.

## Meeting Summary

### Scientific Presentations

*The Development and Framework of MMF as a Regulatory Initiative*

*Liang Zhao, PhD*

*FDA*

In his presentation, Dr. Liang Zhao delved into the development of the MMF framework as a pivotal regulatory initiative designed to enhance drug development and assessment. The MMF initiative represents a strategic effort to leverage the power of M&S tools, which have become essential in the development and evaluation of New Molecular Entities (NMEs) and complex generics.

MMF as a concept has been proposed in the context that M&S tools have significantly advanced the field of drug development. MIDD, which integrates various M&S approaches, plays a crucial role in New Drug Applications (NDAs) and Biologics License Applications (BLAs). The Prescription Drug User Fee Act (PDUFA) VI and VII have formally integrated MIDD paired meeting (pilot) program into the regulatory framework, improving the predictive accuracy and efficiency of drug evaluation processes [27]. In parallel, the Generic Drug User Fee Amendments (GDUFA) III, which launched the MIE Industry Meeting pilot program in October 2023, supports complex generic drug development by addressing significant challenges through advanced modeling techniques [28]. Innovations in Quantitative Systems Pharmacology (QSP) and the integration of Artificial Intelligence and Machine Learning (AI/ML) are pushing the boundaries of personalized medicine, providing more precise and individualized treatment options.

In his presentation, Dr. Zhao illustrated that the core component of the MMF initiative is to further improve model sharing and reusability across drug applications and through the lifecycle management of drugs. By facilitating the reuse of models and reducing redundant efforts in modeling practices, MMFs may help save time and communication costs while promoting a more informed understanding of advancements and benchmark in modeling within the regulatory setting. This framework may not only enhance collaboration among regulatory and industry stakeholders but also break down organizational silos, allowing for the sharing of previous comments and practices on similar regulatory uses. The benefits of MMFs may extend to all parties involved. In the regulatory space, MMFs may enhance regulatory assessment consistency, quality, and efficiency by providing access to previous assessments and promoting a unified approach to M&S. For pharmaceutical industry, MMFs are expected to save the effort of duplicating the models or practices, reduce communication costs with the FDA, and provide enhanced awareness of M&S advancements and their use in regulatory settings. Overall, MMFs may build a transparent and positive ecosystem for the use of models, standardizing practices, and maximizing the impact of M&S approaches, particularly for regulatory use. One of the critical aspects of the MMF initiative is its flexibility in accommodating the dynamic nature of models and data. MMFs are designed to be updated based on the latest knowledge and availability of new data, allowing for multiple models to address the same regulatory need. This approach could encourage scientific publications and the building of venues and platforms for public access to non-proprietary knowledge and information, thereby avoiding the monopoly of stagnant models and fostering continuous innovation.

Dr. Zhao also pointed out that the DMF pathway provides a regulatory mechanism for sharing files of different types, managing the potential downsides associated with file sharing, and realizing benefits for both industry and the FDA [29, 30]. DMFs allow holders to authorize applicants or sponsors to incorporate reference information without disclosing it, facilitating a more streamlined and efficient review process. Multiple DMFs can be used for the same regulatory purpose, and DMF holders can update their files at any time, ensuring that the most current and validated information is always available.

Dr. Zhao concluded that the MMF initiative represents a transformative approach to drug development, enhancing collaboration, consistency, and efficiency among regulatory and industry stakeholders. By breaking down organizational silos, promoting model sharing and reusability, and accommodating the dynamic nature of models and data, MMFs have the potential to set a new standard for innovation and regulatory science. This initiative could optimize lifecycle management across drug applications and incentivize the development of impactful models, driving forward therapeutic advancements and improving public health outcomes. The MMF initiative, supported by robust frameworks like PDUFA, GDUFA, and the DMF pathway, is poised to revolutionize the landscape of drug development and regulatory science.

*Drawing Parallels Between DMFs and MMFs*

*Erin Skoda, PhD*

*FDA*

Considering the audience of diverse backgrounds, Dr. Skoda opened her presentation with an informative definition of DMFs. DMFs are submissions to FDA used to provide confidential, detailed information about facilities, processes, or articles used in the manufacturing, processing, packaging and storing of human drug products. They allow parties to reference material without disclosing DMF contents to those parties, they are not required by statue or regulations, and they are neither approved nor disapproved [29]. There are four types of DMFs [29, 30], and a Type V DMF may be an appropriate vehicle for MMF submissions.

Per the Agency’s current experience based on interactions with stakeholders, while DMFs are not required to be submitted, they do provide benefits to industry, FDA, and patients. DMF assessment often offers efficiency since the DMF may be reviewed to support multiple referencing applications. Additionally, after a DMF becomes adequate to support one application, subsequent assessments by the Agency for a different use or drug product often take much less time. For industry partners, the MMF framework allows for streamlining regulatory submissions when referencing information in a DMF, or a DMF containing an MMF in this case that has already been submitted and assessed by the FDA. The FDA publishes a quarterly list of all active DMFs, which allows for clarity on what may be available to reference by an application [31].

Dr. Skoda explained that DMFs, by nature, have some different operational considerations than applications. A DMF that is submitted to the Agency may be found adequate to support one application and inadequate to support a different application. DMFs offer significant flexibility in referencing since they may be referenced by many different types of applications and even other DMFs. Partial reference of a DMF offers further flexibility. Submitters (DMF holders) should consider formatting and content, which have similar guidelines to applications with additional requirements such as a Letter of Authorization to allow parties to reference the Master File. Changes to a DMF are submitted to the Agency as amendments. These amendments may be administrative or quality amendments and should be accompanied by a summary of changes to clearly indicate what is different from previous submissions.

Dr. Skoda ended her presentation by underlining that the submission flexibility, assessment efficiency, and flexibility in referencing are beneficial aspects of reference to a DMF. It is expected that, while the same principles would apply for an MMF, reference to MMFs will offer similar gains.

*Model Development Lifecycle (MDLC) and Applications in Clinical Development. Implications for MMF*

*Timothy Nicholas, PhD*

*Pfizer*

Dr. Nicholas opened his talk with a thorough description of the MIDD approach and the concept of model development lifecycle (MDLC). MIDD is an approach that uses mathematical and statistical models to integrate data from various sources and inform decision making throughout the drug development process. MIDD can enhance the efficiency and quality of drug development by reducing uncertainty, optimizing study design, and supporting regulatory interactions. However, MIDD may pose challenges, such as the need to ensure the validity, transparency, and reproducibility of the models used, as well as the management and maintenance of the model documentation and code. To address these challenges, MDLC is utilized as a framework to guide the model creation, verification, validation, and reporting activities.

Dr. Nicholas explained that the MDLC concept is based on the idea that models are objects that can be developed, tested, and reused in a systematic and structured way. MDLC consists of four main phases: model specification, model implementation, model verification and validation, and model reporting. In each phase, there are specific tasks and deliverables that need to be documented and reviewed. For example, in the model specification phase, the model objectives, assumptions, inputs, outputs, and hypotheses need to be defined and justified. In the model implementation phase, the model code, data, and parameters need to be developed and checked. In the model verification and validation phase, the model performance, sensitivity, and uncertainty need to be evaluated and compared with alternative models. In the model reporting phase, the model results, assumptions, and conclusions need to be summarized and communicated to the relevant stakeholders. By following the MDLC framework, the model development process can be more transparent, consistent, and reproducible, and the model quality and credibility can be enhanced.

The MDLC concept also enables the use of models as objects that can be stored, shared, and reused in different contexts of use. This is where the MMF framework comes into play. MMF is a term that refers to a collection of model-related documents and files that are organized and submitted to the regulatory agencies as part of the drug development dossier. Dr. Nicholas reasoned that the MMF framework can include the model specification, code, data, verification and validation reports, and other relevant information that support the model objectives and claims. MMFs can also be linked to other parts of the dossier, such as the clinical study reports, the pharmacometric analysis plan, and the summary of clinical pharmacology. By using MMFs, the model development and submission process can be more efficient and standardized, and the regulatory review and feedback can be more streamlined and constructive. MMFs can also facilitate the reuse and update of models for different purposes, such as dose selection, trial simulation, and benefit-risk assessment.

To illustrate his point, Dr. Nicholas shared three potential MMF case studies. The first example was from Purohit *et al*. which utilized a population pharmacokinetic (PPK) model to simulate a healthy participant (HP) cohort within a renal impairment trial [32]. Due to the COVID-19 pandemic, the HP cohort was not recruited. An in-silico cohort was generated based upon the PPK model and, consistent with the analysis of variance approach, demonstrated that a dose modification would not be required. MDLC enabled this methodology by establishing a PPK model early in the development process such that it could be effectively utilized at this time.

Conrado *et al*. described a modeling assessment characterizing Parkinson’s disease progression [33]. This study investigated the use of dopamine transporter imaging, a neuroimaging technique that measures the density of dopamine transporters in the brain, to select patients with early motor Parkinson’s disease for clinical trials. The results showed that this biomarker could differentiate patients with different rates of motor progression, a measure of disease severity and disability, and reduce the sample size needed for trials, a factor that affects the feasibility and cost of research. This example of a ‘deployed’ peer-reviewed MDLC was further presented to the EMA for the enrichment of clinical trials in early motor Parkinson’s disease. This CHMP opinion supported the context of use [34].

The third example summarized disease progression work in Duchenne Muscular Dystrophy (DMD) by Lingineni *et al*. [35]. Here a model-based clinical trial simulation platform was developed to optimize the design, and interpretation, of clinical trials in DMD. This is another example of a ‘deployed’ peer-reviewed MDLC being utilized for a simulation platform. This platform was presented for qualification to the EMA, and while the CHMP did provide a letter of support, further validation was encouraged [34].

**Fig. 1:**
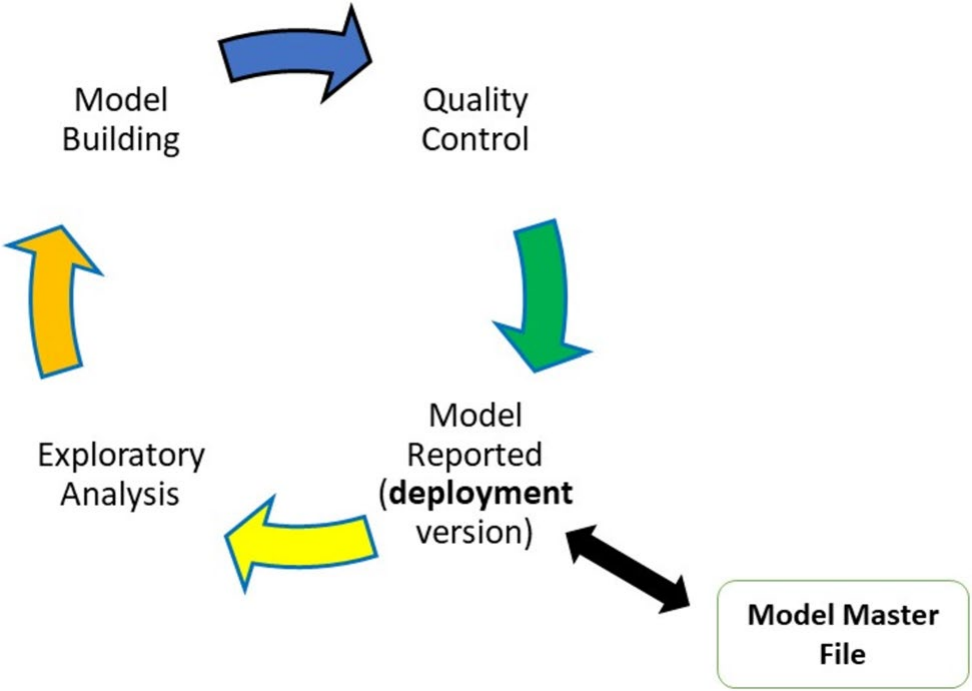
Integration of the MMF concept with the MDLC developed under the MIDD paradigm supporting clinical development of innovator drugs. Presentation titled *Model Development Lifecycle (MDLC) and Applications in Clinical Development: Implications for MMF* delivered by Timothy Nicholas, PhD presented at FDA co-hosted with the Center for Complex Generics (CRCG, https://www.complexgenerics.org) hybrid public workshop titled “Considerations and Potential Regulatory Applications for a Model Master File”.

Each case that Dr. Nicholas presented showed the utility of MIDD in different stages of the MDLC. Each was able to use a model as an object for simulation purposes and finally for discussion with regulatory authorities. In this view, he explained that the MDLC may be considered as a precursor to the MMF. The MMF can be utilized to document a fit for purpose model supporting interpretations and decisions for medicines and novel formulations as shown on Fig. 1.

*Model Master File Framework in Generic Product Development: Challenges, Opportunities and Case Examples*

*Sivacharan Kollipara, MPharm*

*Dr. Reddy’s Laboratories Ltd*

**Fig. 2:**
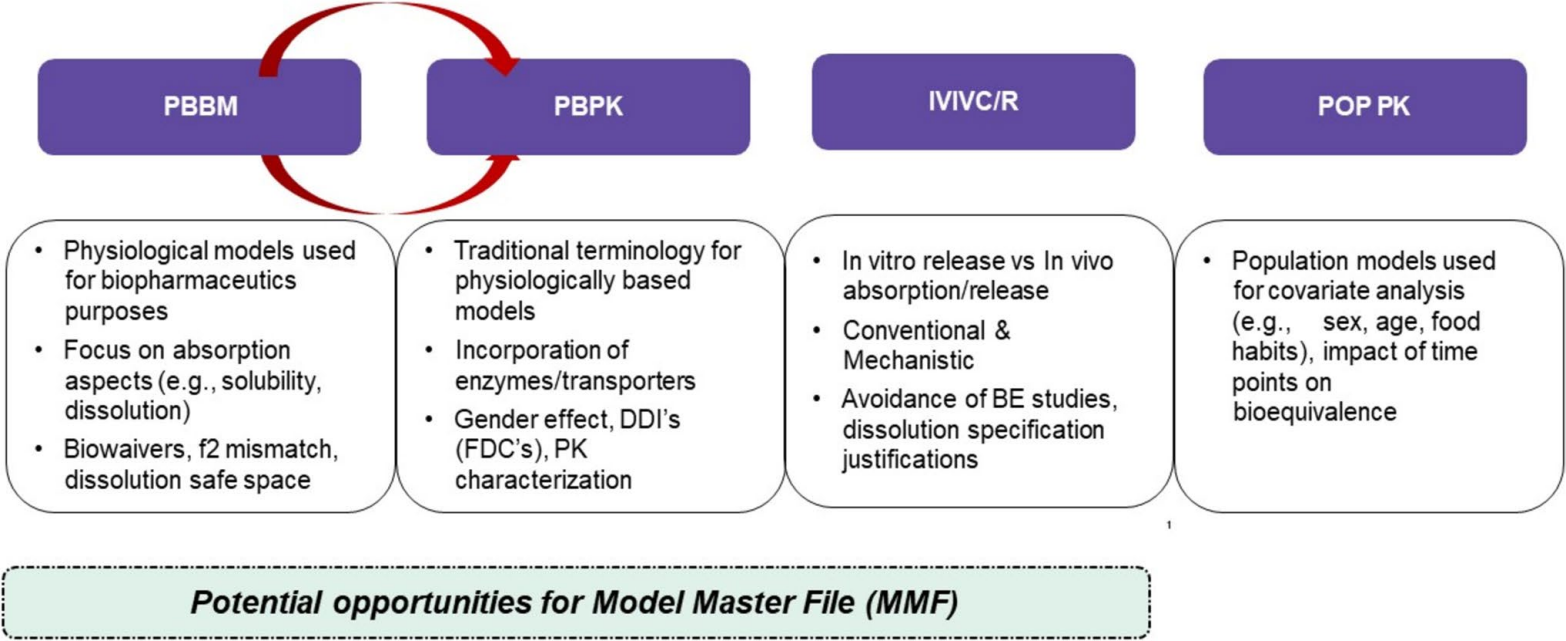
Types of in silico models that are typically used in the development and regulatory submissions of generic drug products. Presentation titled *Model Master File Framework in Generic Product Development: Challenges, Opportunities and Case Examples* delivered by Sivacharan Kollipara, MPharm at the FDA co-hosted with the Center for Complex Generics (CRCG, https://www.complexgenerics.org) hybrid public workshop titled “Considerations and Potential Regulatory Applications for a Model Master File”.

Sivacharan Kollipara delivered the last talk of this session and focused on the potential of the MMF framework in generic drug product development. Applications of PBBM/PBPK in generic drug product development such as BE assessment, biowaivers, justification of dissolution dissimilarity (f2 mismatch), establishing clinically relevant dissolution specifications and dissolution “safe space” were discussed [36]. Various types of models, namely PBPK, PBBM, population PK, IVIVC in the context of generic product development were portrayed. It was indicated that out of all the models, PBPK, PBBM and IVIVC are commonly used models in generic drug development (Fig. 2). Practices related to submission of modeling files to regulatory agencies and commonly received queries on model-based justifications were highlighted.

During this talk, Sivacharan Kollipara made a proposal for MMFs in the context of generic product development. Basic methodology of modeling activities includes components such as the software platform (computational framework, system parameters, open source *vs* non-open source, qualification) and the base model (drug parameters, formulation attributes, pharmacokinetics and physiology). Based on these two components, a model is developed and thus these two components can together comprise an MMF, and once it is established, the MMF can be utilized for various applications. Timelines for the submission of MMF to the agency were also discussed. For instance, conceptually, an MMF could be submitted at any time to the agency, however it would be reviewed only when it is associated with an NDA or an abbreviated new drug application (ANDA). Typically, generic drug product companies use M&S approaches for internal decision making, but a M&S approach within an MMF and associated with an ANDA submission can encourage more applicants to use such modeling approaches effectively. Also, the possibility of integrating MMFs together with MIE program was discussed. The MIE pilot industry meeting program [28] includes three stages: 1) MIE meeting request, 2) meeting preparation & 3) conduct and post meeting communication. During these stages, the concept of MMFs can be raised wherein at step 1, an MMF approach and potential applications can be discussed. Further, during step 2, clarifications and questions on the MMF can be discussed and finally during step 3, FDA and the applicant can discuss potential MMF uses in the prospective ANDA.

Further, generic drug product case examples in the context of MMFs were discussed by Dr. Kollipara. The first case example described the use of a single model for multiple purposes. Initially, the model may be utilized for establishing sex effect on BE assessments but eventually can be applied for establishing the dissolution “safe space” for a product, perform VBE assessments and DDI evaluations. The second case example involved the establishment of dissolution specifications leveraging an in silico model that can be used for BE predictions, establishing “safe space” based on the developed dissolution methodology and assessing the discriminatory power of the proposed *in vitro* dissolution method. The last case example offered by Dr. Kollipara was on a modeling approach developed to establish drug product safety but can have potential applications on critical bioavailability attributes (CBA) evaluation, Scale-up and Post Approval Changes and VBE assessments. In all the case studies, together with the MMF opportunities, model risk evaluations in the context of decision consequence and model influence were performed.

Finally, potential opportunities and challenges of MMF implementation in generic product development were discussed. Dr. Kollipara agreed that the MMF framework can provide opportunities for pharmaceutical companies to interact with the FDA, incentivize the pharmaceutical industry to utilize M&S approaches and reduce timelines in drug development and regulatory approval. MMFs can enhance consistency in regulatory submissions and assessment and enable model reusability. However, potential challenges include unwillingness of applicants to share an MMF publicly, lack of data to validate a M&S approach in an MMF, handling of MMF amendments (e.g., in the case of model versioning) and impact of MMFs on ANDA assessment timelines. Overall, Dr. Kollipara concluded that that the MMF approach appears to be an attractive one in the generic product development space and further understanding is required in streamlining this process.

## Summary of the Panel Discussion

The panel discussion was moderated by Dr. Lanyan Fang (FDA) and Dr. Ke Ren (FDA). The moderators and speakers were joined in the podium by Drs. Stella Grosser (FDA), Bhagwant Rege (FDA), Partha Roy (FDA), Flora Musuamba Tshinanu (EMA) and Hao Zhu (FDA). The panelists had robust discussions on three overarching topics: 1. MMF case studies and applications that they consider as carrying the highest potential for promoting the role of M&S approaches in the space of new drug and generics development, 2. the expected impact and benefit from implementing the MMF framework, and 3. potential pitfalls or unintended consequences from the implementation of MMFs.

The panel discussion opened with Drs. Timothy Nicholas (Pfizer) and Hao Zhu (FDA) acknowledging that the MMF framework is fostering model reusability and, therefore, carries the potential to capture all the knowledge available regarding an NME. In the new drug space, Dr. Timothy Nicholas (Pfizer) discussed potential applications of MMFs, such as a PBPK model developed primarily for waiving a BE study when it encompasses information from several similar compounds that overall increase model credibility. An additional identified MMF application that was discussed is a systems model that may combine several mechanisms of pharmacological activity that have been tested against a variety of carefully selected compounds. In continuing the discussion on potential MMF case studies, Dr. Stella Grosser (FDA) discussed the acceptability within the FDA of frequentists approaches for assessing BE *versus* a Bayesian-based methodology. She recognized the important role M&S approaches play in addressing issues relevant to “outliers” and “single-sex populations in *in vivo* BE studies” that can be benchmarked within the MMF framework. Dr. Grosser invited the panel and audience to consider how the MMF framework could address challenges with drug development in pediatric and other special populations. Dr. Partha Roy (FDA) reasoned that in addition to in silico methodologies and tools, complex bioanalytical methodologies may be considered under the MMF framework. He shared that establishing acceptability of a bioanalytical method could save the industry and regulatory agencies from duplication of effort and lead to overall savings.

Dr. Bhagwant Rege (FDA) and Mr. Sivacharan Kollipara (Dr. Reddy’s Laboratories Ltd.) took the stage to discuss considerations relevant to the MMF framework in the biopharmaceutics space. Dr. Rege opened the discussion stating the MMF framework could support the typically biopharmaceutics-based M&S applications such as mechanistic IVIVCs and PBBM. He pointed out that a discussion on what would constitute an MMF within the scope of these biopharmaceutics M&S applications would increase the clarity on this newly introduced framework. He explained that model components relevant to human physiology and drug substance may be enclosed in an MMF. However, information specific to a formulation would be accessible only to an applicant, who may not necessarily be the MMF holder. As such, Dr. Rege agreed that consistent with the model shareability notion, the MMF concept would undoubtedly save time and resources for industry when developing and applying M&S approaches while allowing for efforts targeting model development based on drug product-specific parameters and model verification and validation (V&V) against its “context of use”. Mr. Kollipara discussed one of the key building blocks of biopharmaceutic models, biopredictive *in vitro* methods. He stressed that the manufacturing process impacts these methodologies significantly and this poses a challenge for generic manufacturers. He further mentioned that in majority of the cases, the quality control (QC) media is not biopredictive and the impact of formulation or process variables evaluated through QC media may not provide clinical impact of these variables. He acknowledged the tremendous amount of knowledge and studies that are crystallized into these types of methodologies that are then leveraged for developing mechanistic in silico models. Considering the MMF framework protects any proprietary information, he was confident that it could be a framework for capturing this accumulated knowledge in a comprehensive manner and establish credibility for PBBM applications. The later would benefit multiple regulatory applications and improve regulatory assessment consistency.

Speakers and panelists focused then on how the MMF impact can be maximized and considered the potential benefits associated with the MMF initiative. Dr. Liang Zhao (FDA) explained that from an operational aspect, implementation of the MMF framework would increase efficiency in applying M&S approaches for the pharmaceutical industry and efficiency in assessing regulatory submissions with M&S approaches. Dr. Zhao further discussed that the MMF could promote “best practices” for M&S methodologies and applications and serve as a framework for the development of benchmark M&S tools and applications that can be utilized to promote the role of M&S, streamline regulatory submissions and regulatory assessments, and identify potential gaps and roadblocks in establishing M&S applications for decision making. Dr. Zhao offered his opinion on the opportunity the MMF framework holds for industry partners that may not have the resources for conducting an *in vivo* study when this study may be waived by a referenced MMF in their regulatory submission and for pharmaceutical companies that do not have internal capability for developing M&S approaches. Dr. Zhao underlined that the MMF framework improves accessibility to M&S approaches for industry and offers the opportunity for applying the same or several validated, for their intended purpose, versions of a model across different applications in the innovator and generic drug space while safeguarding proprietary information. Dr. Zhao noted that the MMF initiative can further incentivize the development of new in silico models or expand the use of those that have already gained acceptability under the “context of use” consideration. He concluded that ultimately, implementation of the MMF initiative is a step towards establishment of M&S approaches in the drug development paradigm and in regulatory decision making.

Dr. Partha Roy agreed that the MMF framework could foster efficiency and consistency in regulatory assessment. He shared the view that an M&S approach submitted under an MMF could potentially support an ANDA when the reference standard is not available in the market. Dr. Roy noted that MMFs with V&V activities for models that integrate a wealth of knowledge on a drug product and account for inter-study and other sources of data variability may lead to faster regulatory assessment and improve the regulatory acceptability of in silico approaches. Considering the increasingly important role of M&S applications in regulatory submissions in the new drug and generics space, it is expected that MMFs delving into aspects of model V&V and serving as benchmark cases would result in more efficient and targeted regulatory assessments informing regulatory decision-making.

From the EMA, Dr. Flora Musuamba Tshinanu offered insightful comments on the MMF initiative based on the EMA experience with the Qualification of Novel Methodologies for Medicine Development [37]. She recognized the added value the MMF framework offers to assessors making them aware of previously evaluated versions of a M&S application which is a critical factor for regulatory assessment consistency. She reiterated that the MMF proposal may assist with benchmarking in silico methodologies, tools and applications and increase their acceptability by regulatory agencies and pharmaceutical industry. However, she noted the need for a clear description of the “context of use” for an M&S application captured in an MMF will ensure that model credibility is appropriately established between regulatory applications. Dr. Tshinanu stressed that when new knowledge, i.e., data, is generated on a drug product or when the question of interest for the developed model is modified, the content of the MMF and its overall “context of use” statement should be revised to reflect these changes and called for further discussion on this topic. In agreement with Dr. Tshinanu’s comment, Dr. Erin Skoda (FDA) explained that a DMF can be considered “adequate” only for its intended purpose. To the extent Type V DMFs are used for the submission of MMFs, the “context of use” of a submitted MMF would be expected to be clearly stated by the DMF holder and clearly noted by the applicant referencing such DMF.

Finally, the panel considered potential unintended consequences and pitfalls for the MMF framework. The panelists pointed out the diversity of stakeholders involved in the “ecosystem” of drug development including new drug industry, pharmaceutical companies for generics, consulting companies specializing on in silico methodologies and tools and academia. As such, it is challenging to foresee how the MMF framework implementation would impact the business model for each one of these interested parties. Protection of intellectual property and proprietary information remained a concern for pharmaceutical industry partners as noted by Drs. Nicholas and Kollipara. From a regulatory perspective, there was agreement that the MMF framework holds a great potential for advancing the role of M&S approaches and increase regulatory assessment consistency and efficiency. However, as Drs. Nicholas and Tshinanu pointed out, it may be challenging for sponsors/applicants and FDA to determine the adequacy of an MMF across different regulatory applications considering the “context of use” stated for a referenced MMF. The panel agreed that within the scope of implementing the MMF framework, further discussion and close consideration is required on how testing for certain model assumptions, accounting for drug product-specific information into a proposed model and performing fit-for-purpose V&V to support model credibility would supplement a M&S approach supporting a regulatory application when an MMF is referenced.

## Small Group Discussion Session

During the small group discussion sessions at the end of Day 1 and 2 of the workshop, the workshop attendees developed their thinking and engaged in fruitful discussions on the following topics: what are the key considerations when developing an MMF (content and format), what are the potential incentives and benefits associated with the development of MMFs, how can all interested parties maximize the benefits from implementing the MMF framework, MMF lifecycle management and other operational considerations. Finally, the session allowed for the FDA to collect valuable feedback on the challenges and unintended consequences industry parties have identified. A summary of the key points of the in-person discussions are available in the Supplementary Material section.

## Conclusions

This report summarizes the proceedings of Session 1/Day 1 of the DDA-CRCG co-hosted public workshop “Considerations and Potential Regulatory Applications for a Model Master File” that took place on May 2 and 3, 2024. The session attendees thoroughly and systematically discussed the MMF initiative and worked on understanding the implementation parameters of the MMF framework under the DMF umbrella. Subject matter experts from the industry, academia, Contract Research Organizations, consulting companies and regulatory agencies explored pathways in the innovator drug and generics space through which the MMF framework can promote the role of M&S approaches in supporting decision making on drug product development and approval paradigm.

## Supplementary Information

Below is the link to the electronic supplementary material.

Supplementary file1 (DOCX 44 KB)

## Data Availability

Data sharing is not applicable to this article as no datasets were generated or analyzed during the current study.
