# Supplementary material for: Considering Opportunities and Challenges When Implementing the Model Master File Framework – a Meeting Report
Source: Pharm Res. 2025 Mar 7;42(5):737–46. doi: 10.1007/s11095-025-03839-x (PMC12158848; doi:10.1007/s11095-025-03839-x)
Supplement: Supplementary file 1 — Supplementary file1 (DOCX 44 KB) [file 11095_2025_3839_MOESM1_ESM.docx]

Considering Opportunities and Challenges When Implementing the Model Master File Framework – a Meeting Report

Eleftheria Tsakalozou, Lanyan Fang, Erin Skoda, Timothy Nicholas, Sivacharan Kollipara, Ke Ren, Stella Grosser, Bhagwant Rege, Partha Roy, Hao Zhu, Liang Zhao

Conflicts of interest

The opinions expressed in the manuscript are those of the authors and should not be interpreted as the position of their organizations/employers. The contents of the manuscript are those of the authors and do not necessarily represent the official views of, nor an endorsement, by FDA/HHS, or the U.S. Government.

Supplementary Material

**FDA co-hosted with the Center for Complex Generics Public Workshop**

**“Considerations and Potential Regulatory Applications for a Model Master File”**

**May 2-3, 2024**

**Summaries of the Small Group Discussion Sessions**

**Key considerations when developing a Model Master File (MMF)**

**What is an MMF (develop a definition), what are the type of MMFs and what type of (in**

**silico) models could be considered MMFs?**

The workshop attendees worked together in distilling their improved understanding of the Model Master File concept into succinct and informative definitions on the MMF concept. The MMF definitions that were documented include:

- “MMFs are submissions to FDA that may be used to provide confidential, yet detailed information about model equations, scripts, model development process and/or model qualification to support a specific regulatory decision”, provided by Dr. Joga Gobburu.
- “MMF refers to a quantitative model **and/**or a modeling platform that has undergone sufficient model Verification & Validation to be recognized as sharable intellectual property that is acceptable for regulatory purposes.” This suggested change (**bold** text above) is to reflect the fact that a model may be an essentially part of a modeling platform and thus inseparable from it.
- “MMF is a framework designed to view in silico models or methodologies/practices associated with these models as portable, reusable, generalizable, and sharable, following comprehensive Verification & Validation (V&V).”

The attendees agreed that characteristics that render a modeling and simulation application a good candidate for being submitted as an MMF include but are not limited to: reusability and repeatability across different sponsors, or for the same sponsor across different products, or for the same product throughout its lifecycle supporting technology transfers, regulatory approval and product post approval changes. An MMF could also be utilized to address global harmonization challenges.

Types of MMFs were considered based on the methodologies that support modeling approaches. For instance, these can be:

- Physiologically based pharmacokinetic (PBPK) models
- Physics-based models (e.g. computational & CFD models)
- Artificial Intelligence (AI) models
- Process engineering and Manufacturing models
- Statistical models
- Quantitative Systems Pharmacology (QSP) models

Types of MMFs were identified also based on the model application or model structure characteristics. These models include physiologic/disease progression models, active ingredient (e.g., disposition kinetics) models, product specific (e.g., immediate or modified release oral dosage forms) and complex formulation models (e.g., long-acting injectable drug products), models of virtual organs or organ tissue or tumor, drug-drug interaction (DDI) models. Additional examples of potential MMFs following this type of categorization may include models capturing in vitro-in vivo relationships (IVIVR), utilizing allometric scaling factors to predict PK parameters, and models of Alzheimer’s disease. These models could be applied across different drug products exemplifying their utility as MMFs. Finally, an MMF can be developed at the level of a modeling framework (platform), module, model, and parameters. Finally, an MMF can encompass a methodology such as determining the appropriateness of in vitro data for the establishment of an IVIV extrapolation (IVIVE) methodology.

**What are the key components of an MMF regarding its preparation, validation and submission?**

Several key considerations for the development of a MMF were discussed such as the MMF resolution (e.g., broad platform or single, specific case), purpose of the MMF and whether it may be accepted for another purpose, the methodology for its use, and the data required to validate the model.

The attendees noted that MMFs may vary in terms of content and format depending on the MMF type. A standard format for documenting the model/modeling approach, such as a template, may be needed. The template can be simple but should contain all the required details, described as follows. The MMF template should include a context of use (COU) within the regulatory submission, which will dictate, among others, how stringent the model validation procedure should be. The MMF should also include the title and type of file(s), the scientific rationale for the model, any modeling assumptions, model diagnostics, all major model development steps, validation/verification, the model’s intended application(s), and any associated publications; however, the content will vary depending on the type of model and may not need to include observed data. The MMF may also contain all the raw data files used for model building, validation/verification and application. This description was closely aligned with the two MMF examples the workshop organizing committee had developed and distributed prior to the workshop.

Potential applications of MMF for a specific COU is a critical component of the MMF. Clear definition of how the model will be applied is key for the development and regulatory assessment of an MMF. The participants emphasized the clarity of reporting the development and validation process. The MMF may contain the underlying code, equations and any assumption in model development process. Model validation is key to the development of MMFs that would include a model and was extensively discussed. It was agreed that the consequence of a regulatory decision informed by a model will indicate how mechanistic the model should be (i.e. if the model accounts for all physiological process accurately), the appropriate data input, the appropriate software platform version. Additionally, the validation for a model informing a risk-based assessment could be less extensive compared to the validation of a model informing a regulatory decision or replacing a pivotal study. However, regarding the MMF context of use, the challenge of developing an MMF specific enough to be applied to the intended purpose, but also flexible enough to apply to different contexts of use within that purpose was acknowledged. In the context of MMFs, model validation is the key as it may be used for multiple future applications.

It was also suggested that the MMFs could be divided based on open source and non-open-source information. It was noted that there may not be an incentive to make MMFs open-source until after drug product approvals or publication of the M&S work described in the MMFs. However, open source MMFs would deviate from the basic operating principles of a DMF.

The attendees stressed that a communication path with FDA or a published guidance could help outline expectations for a MMF submission with respect to content, format, and validation. Further discussion is necessary for successful MMF submissions.

**What are the key considerations in the legal and/or financial aspects related to MMFs?**

On the legal/financial aspects of the MMF implementation, discussions revolved around safeguarding proprietary and confidential information within an MMF submission. Industry representatives explained that it is critical for them that measures are in place protecting proprietary information and drug product specific parameters. In their opinion, this may be challenging considering the contents of the MMF need to be known to the applicant that references it.

Due to the sharable nature (within the context of a letter of authorization) of the MMF, under a mutually beneficial relationship, the workshop attendees agreed that additional discussions on the confidentiality of data may need to take place. The nature of these discussions is not foreign to the interested parties; CROs operate within a similar environment where they communicate with sponsors/applicants and utilize proprietary information towards model development and validation.

The attendees recognized that the business component behind the MMF framework is largely unknown and hard to predict considering parties from the innovator drug side, generics and contract research organizations and consulting companies are involved. Naturally, time and resources saved using an established MMF may serve as the basis for negotiation for MMF access and sharing. However, understanding the cost implications of developing and maintaining MMFs and managing intellectual property issues is an important parameter when implementing the MMF framework.

**What are the potential benefits/incentives for stakeholders to develop and use an MMF?**

**How may the MMF framework to enhance regulatory approval for product portfolios supported by M&S approaches?**

For innovator companies, the creation and use of an MMF could be beneficial for follow-on products, for extending a product to special populations and rare disease populations, for disease progression models, DDIs, combination therapies, etc. MMFs may also be useful in facilitating the application of modeling and simulation approaches informing early phase trials, dosing decisions in later trials, waiving food effect studies, support decision making for formulation changes, for bridging populations, and new label extensions. When specific to a drug product, an MMF could help with lifecycle management of said product. An MMF can provide additional evidence that would not have to be re-reviewed as intensively as a newly submitted model, thus saving review time and de-risking utilizing a modeling and simulation application in support of a regulatory submission. Overall, the MMF may lead to reduced development and operational costs by leveraging shared and validated models and through more efficient data management and utilization.

The use of MMFs may be significantly beneficial to generic companies, particularly those with in-house modeling and simulation capabilities. The use of an MMF can increase the confidence into the product development and can enable faster generic product approval. A potential advantage of an MMF owned by a generic company is that it is built on the accumulating knowledge of the specific drug substance or the reference standard which could potentially increase the amount of data available for model development and validation.

Consulting companies and other CROs could have greater flexibility in applying modeling approaches across several drug products when utilizing the MMF framework. Within that framework, MMFs could allow sharing of developed modeling frameworks and models and therefore, reduce duplication of effort and foster shared understanding on the development of challenging products. This may overall increase efficiency in the product development process and reduce associated costs.

Across all interested parties, the MMF framework can facilitate clear and efficient communication among software companies, academia, and regulatory agencies, supporting global harmonization efforts. The adoption of an MMF by one regulatory agency may help streamlining the review and approval processes by other agencies although this pathway may need to be explored further.

**How would an MMF streamline a regulatory submission?**

From the regulatory perspective, a MMF can result in productivity increase, efficiency enhancement and can enable consistency in regulatory review. By implementing the MMF framework, regulatory agencies may benefit from streamlined review processes, leading to reduced review times. This may be increasingly important considering the restrictive PDUFA and GDUFA review timelines. Finally, an MMF found acceptable within a previous regulatory submission would not need to be re-assessed in future regulatory submissions referencing it, apart for its context of use. This may lead to decreased regulatory assessment times and increased regulatory consistency. MMFs potentially linked with the MIE initiative can enable the development and regulatory approval process for complex drug formulations.

**How would an MMF increase regulatory acceptability of modeling and simulation approaches?**

MMFs could help remove regulatory risk of including a modeling and simulation approach in support of a regulatory submission as the same model, under an MMF found adequate for its context of use, could be used along the entire life cycle of the drug product. For instance, for both new drug development and generics, the use of innovator IVIVC models may be used to support biowaivers under manufacturing change scenarios. In addition, an MMF may provide a standard methodology for certain model applications (e.g., virtual clinical trial simulation or virtual bioequivalence assessment). An MMF, when found adequate, may serve as a centralized mechanism for review and recommendations from regulatory agencies.

**Maximize the Benefit from Implementing the MMF Framework:**

**Which type of products or therapeutics areas may benefit the most from MMF applications?**

The workshop participants agreed that every therapeutic area or dosage forms can benefit from MMFs. Products or therapeutic areas where interested parties have significant experience and established confidence in using modeling and simulation approaches stand to benefit the most. This familiarity enhances the effective implementation and optimization of MMFs. To be more specific, M&S approaches that may support alternative BE approaches for LAIs/OIDP, locally acting, anticancer drugs or other complex injectables may benefit from the MMF framework.

Both generic and new drug developers can equally utilize MMFs for their drug development and approval process. Systemic products, particularly oral dosage forms and those involving drug-drug interaction (DDI) and disposition models for complex generics, are likely to see considerable benefits from MMF applications. These benefits arise, in part, from the framework’s ability to streamline and standardize validation processes. MMFs can minimize the effort and resources to build the model since the same MMF or part of an MMF can be reused across multiple applications. LAIs may additionally benefit with the concept of MMF (potentially linked with MIE) due to the lack of generics. Products where a M&S approach can be utilized to address issues with a food effect or rare diseases where a M&S approach can help overcome challenges with the development of these products.

QSP models may benefit from the MMF framework. QSP models are large models, complex and their validation is challenging. However, as knowledge is increasing during the development program for a new drug entity, the confidence on a QSP model (disease model, for instance) is increasing as well. When this model is tested across multiple molecular entities, the establishment of an MMF for this disease model would include a comprehensive validation process. Furthermore, a disease progression or disease progression with standard of care model would be ideal MMF cases that could be used by an applicant for life cycle management of their product or across different applicants for their products that may be relevant to the specific disease model.

**What specific considerations would apply to MMFs based on the delivery route of the drug product of interest?**

The attendees agreed that the MMF framework may address model validation issues for models for both oral and non-oral dosage forms when supporting bioequivalence assessments, particularly in scenarios lacking non-bioequivalent (BE) batch data, by providing a robust framework for consistency and repeatability in model validations. Alternatively, MMFs may support modeling and simulation approaches aimed at mitigating risks associated with not conducting certain studies, such as fed studies and PPI interaction studies, by leveraging validated modeling predictions to infer outcomes.

**How could an MMF be applied to support modeling and simulation approaches on the development of innovator drugs or alternative bioequivalence approaches for complex generics?**

In the innovator drug space, the development of a drug product specific model is accompanying the drug product development program at every step of clinical development (phase 1, 2, 3 clinical studies) incorporating knowledge gained incrementally. This model is utilized not only to support approval but throughout the life cycle of the product for post approval changes such as manufacturer changes. At the late stages of the product development program, this mature model may only require minimal changes to be applied and therefore could be submitted as an MMF. As an MMF found to be adequate and not requiring regulatory assessment with every submission, it may streamline regulatory submissions and reduce regulator assessment times. A systems model that can predict effect of acid reducing agents was discussed as an MMF example in the new drug space.

For generics, the participants agreed that complex products (including locally acting drug products) may benefit more from MMFs. Developing an MMF to support approval of the first generic (ensuring 6-month exclusivity) or a first in class therapeutic in the innovator side is a major incentive for industry. Along those lines, referencing an MMF that has already been found adequate for its intended purpose is a step towards de-risking product development supported by M&S approaches for generics. Additionally, an MMF that can be utilized to support multiple generics referencing the same reference standard may facilitate the development and approval of generic drug products by incorporating accumulating knowledge across many products into a single model application, reducing regulatory assessment times and, increase regulatory acceptability. Such an MMF may be leveraged for first generic approval, but also for lifecycle management of any generic drug product (e.g. SUPAC changes). The same MMF can be leveraged across similar products within the pipeline facing similar challenges. The attendees recognized the important role consulting companies and CROs with M&S expertise may play under these scenarios.

**MMF lifecycle management**

**How is the dynamic nature of in silico models handled when submitted as MMFs?**

Modeling and simulation advancements are driven by progress in the field of computational science and improved understanding of the biology/pharmacology and drug product/device characteristics that the models are describing. Advances in scientific understanding or new research findings necessitate updates on the developed models to maintain relevance and accuracy. The models submitted under an MMF cannot be an exception. In the computational side, changes in modeling software might require an updated model to maintain compatibility.

**What would be the mechanism or criteria for MMF revision (amendment) or a new MMF submission?**

The discussion group suggested that a key consideration of updating (amendment) or submitting a new MMF would be a change in the context of use for an MMF. However, in concept, applicants may choose to reference an MMF subsection, even if it is not the most recent one (similar to DMFs), if appropriate for its context of use. Any update to an MMF should include a detailed account of assumptions which prompted changes to the model, any changes to the model equation(s), and a succinct description of the reason for any changes to the model and any impact on model assumptions.

The need for FDA input on the level of change that would necessitate a new MMF was discussed amongst the attendees. Per the discussion, it would be helpful for regulatory agencies, such as FDA, to develop pre-screening criteria for MMF submissions that would provide guidance to prospective MMF holders on this issue. Regarding the operational aspect of an MMF amendment, this amendment is expected to be communicated by the MMF holder to all affected parties per the principles that apply to all DMFs.

PBPK modeling frameworks (software) are being regularly updated as knowledge and evidence becomes available and leads to more mechanistic understanding of a process that is relevant and captured in the model. These changes that are essentially changes in the structure and relevant assumptions on the model may trigger an MMF amendment or a new MMF if the context of use is changing. Representatives from software developer companies explained that when a new software version is released, it is extensively tested for backward compatibility. Additional considerations may apply in the case where a model described in an MMF is not modular or is part of the modeling framework. Depending on the context of use, additional documentation may be necessary to capture potential changes in the model performance as a result of changes in the modeling framework under a new software version.

Population pharmacokinetic (POP PK) models have their own considerations on MMF versioning. However, based on the discussions, software versioning may not trigger an MMF amendment in all cases because the qualification/validation process of POP PK models is extremely standardized and therefore, to some extent, can be independent of software version.

**What additional input on the MMF mechanism should the FDA consider?**

One of the key challenges with the implementation of MMFs facing the pharmaceutical industry, innovator drugs and generics, pertains to patent protection, and protection of proprietary and other confidential information within the boundaries of the MMF. It was clarified during the workshop discussions that the MMF framework would be implemented under the same guiding principles as DMFs under which proprietary information is safeguarded by the MMF holder.

The participants noted their appreciation for the available general guidances on the development and validation of in silico models as these guidance documents would be helpful in the MMF building process. However, prospective MMF holders may still have additional questions on the MMF qualification process. The attendees pointed that a public dialogue between FDA and MMF holders may be helpful. The attendees discussed the need for a system to pre-screen an MMF before it would be associated with a regulatory application. It was noted that the FDA’s expectations on the evaluation of an MMF and its potential applications within M&S approaches supporting regulatory submissions could be communicated through workshops, conferences, or publications.

The workshop attendees agreed that it would be beneficial to:

- Maintain a publicly available database of MMFs that would provide information on the MMF holder and the MMF context of use at minimum, similarly to the annual DMF report^[[1]](#footnote-2)^. Such a database would enhance transparency and accessibility for all stakeholders and promote broader adoption and trust in the MMF framework.
- Make the public aware of any MMFs that have been found adequate after being referenced in a regulatory submission. These case examples will assist with communicating the benefits of MMFs.
- Conduct research, if necessary, to promote the implementation of MMFs.
- Develop a general guidance on the MMF framework that would provide a roadmap on the development of MMFs, outline the FDA’s expectations and clearly describe the operational aspects of the MMF framework.
- Develop a communication path with the FDA that prospective MMF holders could use to engage with the FDA on the MMF framework.

**Acknowledgments:**

For documenting and summarizing the discussions that took place during the Small Group Discussion Sessions, the workshop organizing committee would like to express their sincere gratitude to: Drs Khondoker Alam, Yi-Hsien Cheng, Ross Walenga, Jae Lee, Fang Wu, Arindom Pal, Yuqing Gong, Robert Hopefl, Eleftheria Tsakalozou, Steven Chopski, Lanyan Fang, Mark Donnelly, Liang Zhao, Natalie Jusko, Mingliang Tan, Rebecca Moody.

1. <https://www.fda.gov/drugs/drug-master-files-dmfs/list-drug-master-files-dmfs> [↑](#footnote-ref-2)
